# Untargeted metabolomics reveals the metabolic basis of sugar–acid balance and quality differentiation in melon

**DOI:** 10.3389/fpls.2026.1853789

**Published:** 2026-06-19

**Authors:** Jiayue Wu, Bin Xu, Haojie Yao, Xiusi Yang, Yang Liu, Guanming Chen

**Affiliations:** 1 Innovation Team of Southern Breeding Technology, Culture and Strategic Research, College of Breeding and Multiplication, Hainan University, Sanya, China; 2 College of Tropical Agriculture and Forestry, Hainan University, Haikou, China; 3 Sanya Academy of Tropical Agricultural Sciences, Sanya, China

**Keywords:** *Cucumis melo* L., fruit metabolomics, fruit quality, organic acids, untargeted metabolomics

## Abstract

Melon fruit quality is shaped by the combined effects of sugar–acid composition, texture, antioxidant-related traits, and diverse soluble metabolites. In this study, two tropical netted melon accessions with a close genetic background but contrasting quality-related phenotypes—melon 109 and its sister line 108 (CK)—were compared. Physicochemical analysis, antioxidant-related assays, and untargeted UPLC–MS/MS metabolomics were used. Melon 109 showed higher flesh firmness, total soluble solids, titratable acidity, and antioxidant-related enzyme activities, but a lower sugar–acid ratio and malondialdehyde content than CK. Untargeted metabolomics detected 2101 metabolites and identified 246 differential metabolites, including 146 upregulated and 100 downregulated metabolites in melon 109. These differential metabolites included carbohydrate-related derivatives, organic-acid-related compounds, amino-acid-derived metabolites, and specialized metabolites, suggesting that quality differentiation was not driven solely by simple sugars or individual organic acids. KEGG enrichment analysis further highlighted amino sugar and nucleotide sugar metabolism, biosynthesis of nucleotide sugars, and terpenoid backbone biosynthesis. These pathways may provide putative pathway-level clues related to sugar-derived metabolism, nucleotide-sugar-associated structural metabolism, terpenoid precursor metabolism, and antioxidant-related quality traits. Overall, this study suggests coordinated metabolic remodeling associated with the distinctive quality profile of melon 109 and provides candidate metabolite classes and pathway-level information for quality-oriented melon breeding.

## 1 Introduction

Melon (*Cucumis melo* L.) is an economically important crop worldwide and belongs to the family Cucurbitaceae. During domestication, melon fruit flavor has shifted from small, sour, and bitter types to larger, sweeter fruits with low acidity and thick, fleshy pulp (Zhao et al., 2019; Lyu et al., 2023). The currently prevalent high-sugar, low-acid flavor profile of melon fruit reflects long-term human selection (Paris et al., 2012; Cohen et al., 2014; Lyu et al., 2023). Melon is also recognized as a valuable source of vitamin C, β-carotene, dietary fiber, and diverse phytochemicals, including free and bound phenolic compounds. These constituents exhibit antioxidant-related properties *in vitro* (Wang Y. et al., 2023; Silva et al., 2025) and may contribute to health benefits by helping to mitigate oxidative stress through free-radical scavenging. As living standards continue to rise, consumer demand is increasingly shifting toward high-quality melons with more diverse flavor characteristics (Yu et al., 2024). Preference for melon fruit has gradually evolved from sweetness alone to a more integrated appreciation of sugar–acid balance and aroma profiles (Escribano et al., 2010).

Untargeted metabolomics is a comprehensive analytical approach that uses high-resolution mass spectrometry to profile a broad range of metabolites in biological samples without predefined targets (Doğan, 2024). It enables systematic characterization of metabolic variation and facilitates the identification of metabolic pathways and molecular features that may be overlooked in targeted analyses (Kaczmarek et al., 2023). This strategy has been widely applied in studies of fruit development, ripening, and cultivar comparison. For example, untargeted metabolomic analyses have revealed substantial differences in flavor-related compounds, such as sugars and organic acids, among melon cultivars (Moing et al., 2020). Recent metabolomic and transcriptomic studies have shown that melon fruit quality is closely related to changes in primary metabolism. These changes mainly involve organic acid accumulation, firmness-associated metabolism, and metabolic regulation (Zhang et al., 2023; Shao et al., 2024). Previous melon metabolomic studies have mainly emphasized primary metabolites and pathways directly related to common quality traits, such as sugars, organic acids, and amino acids changes. However, less attention has been paid to sugar-derived metabolic routes and specialized metabolism-related processes that may connect primary carbon metabolism with fruit quality formation. In this study, KEGG-based pathway analysis highlighted several related pathways, including amino sugar and nucleotide sugar metabolism, biosynthesis of nucleotide sugars, and terpenoid backbone biosynthesis. These pathways were therefore further examined to explore their potential links with sugar–acid composition, nucleotide-sugar-mediated structural remodeling, aroma precursor supply, and other quality-related traits in melon 109.

Melon 109 and its sister line 108 (CK), two tropical netted melon accessions with a close genetic background but contrasting fruit quality-related phenotypes, were selected as comparative materials. Preliminary breeding evaluation indicated that these two accessions differed markedly in sugar–acid composition and antioxidant-related traits, providing a suitable system for investigating metabolic changes associated with compositional quality differentiation. We hypothesized that the distinctive quality profile of melon 109 is not solely attributable to changes in simple sugars or individual organic acids, but is associated with coordinated remodeling of multiple soluble metabolites and related metabolic pathways. Untargeted metabolomics was then applied to characterize differential metabolites associated with these traits. KEGG-based pathway analysis was subsequently used to identify metabolic routes potentially related to sugar–acid composition, nucleotide-sugar-associated structural metabolism, terpenoid precursor metabolism, and antioxidant-related quality attributes. This study aimed to clarify the metabolite classes and pathway-level features underlying the distinctive compositional quality of melon 109, thereby providing biochemical insights into fruit quality differentiation and a metabolic basis for quality-oriented melon breeding.

## 2 Materials and methods

### 2.1 Materials and sampling

**Figure 1:**
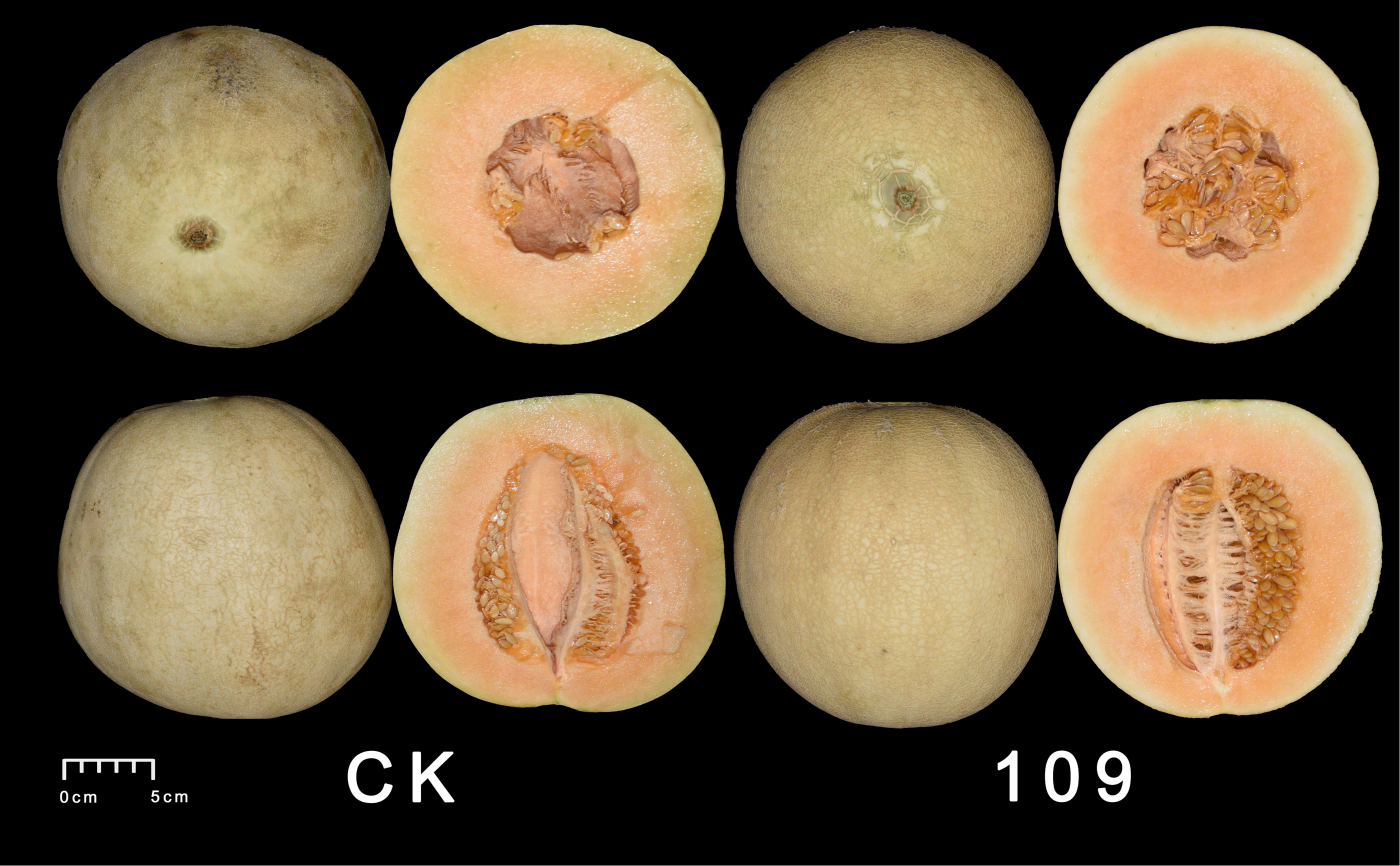
The mature fruit of CK and 109 melons.

As illustrated in **Figure 1**, mature fruits of genotypes CK and 109 were harvested from the Sanya Melon Science and Technology Backyard, Hainan, China (18.3156° N, 109.4755° E). Commercial maturity was determined by days after pollination (DAP) alongside external horticultural traits. For each genotype, three biological replicates were prepared, each comprising pooled tissue from six individual fruits. Samples were immediately snap-frozen in liquid nitrogen and homogenized for further analysis.

### 2.2 Basic qualities analysis

The longitudinal and transverse dimensions of the fruit were assessed with a tape measure, and the ratio was computed to determine the fruit shape index. Fruit weight was determined using an electronic analytical balance. Flesh hardness was measured using a fruit hardness tester (Aipli GY-3, Zhejiang, China). The flesh thickness was determined using a caliper. Soluble sugar content was determined by the anthrone–sulfuric acid colorimetric method. Absorbance at 620 nm was measured with a visible spectrophotometer (INESA 722N, Shanghai, China). Soluble sugar content was determined via the anthrone–sulfuric acid colorimetric assay and expressed as glucose equivalents. The concentration was calculated based on the calibration curve y = 0.0074x + 0.0011 (R² = 0.9979). The titratable acidity (TA) was assessed through a titration process utilizing 0.1 mol/L NaOH, following the guidelines set by GB 5009.239–2016. To determine the total soluble solids (TSS), a handheld refractometer (PAL-1, Atago, Tokyo, Japan) was employed.

### 2.3 Antioxidant analysis

Ascorbic acid content was determined using the 2,6-dichlorophenolindophenol titration method according to GB 5009.86–2016. Malondialdehyde (MDA) content was quantified by spectrophotometry following GB 5009.181–2016. Peroxidase (POD) activity was assayed using the guaiacol method in accordance with GB/T 32131–2015. Catalase (CAT) activity was measured by the potassium permanganate titration method according to GB/T 5522–2008. Superoxide dismutase (SOD) activity was determined by spectrophotometry following GB/T 41906–2022.

### 2.4 Untargeted metabolomics analysis

A 30 mg aliquot of the sample powder was accurately weighed and extracted with 1,500 μL of 70% aqueous methanol containing dichlorophenylalanine as the internal standard at a final concentration of 250 μg/mL, pre-chilled at −20 °C. During extraction, the samples were vortexed for 30 s at 30 min intervals, and this procedure was repeated six times. The extracts were then centrifuged at 12,000 rpm for 3 min, after which the supernatant was collected, filtered through a 0.22 μm microporous membrane, and transferred into injection vials for subsequent UPLC–MS/MS analysis.

Metabolite identification and quantification were performed by a UPLC–MS/MS platform (UPLC: Thermo Scientific Vanquish, MS: Q Exactive HF-X, Thermo Scientific, Massachusetts, USA). Chromatographic separation was performed using a Waters ACQUITY UPLC HSS T3 column (1.8 μm, 2.1 mm × 100 mm). The column temperature was maintained at 40 °C, with a flow rate of 0.4 mL/min. A 4.0 μL aliquot of the sample was injected for analysis. The mobile phase consisted of ultrapure water containing 0.1% formic acid (solvent A) and acetonitrile containing 0.1% formic acid (solvent B). The gradient elution conditions of the mobile phase are shown in **Table 1**. The chromatographic effluent was directed into a mass spectrometer and monitored in-line by electrospray ionization (ESI). The mass spectrometric conditions for the Q Exactive HF-X are shown in **Table 2**.

**Table 1:** The gradient elution conditions of the mobile phase.

Time (min)	A (%)	B (%)
0.0	95	5
5.0	35	65
6.0	1	99
7.5	1	99
7.6	95	5
10.0	95	5

**Table 2:** Q Exactive HF-X mass spectrometry conditions.

English name	ESI+	ESI−
Spray Voltage (V)	3500	3200
Sheath gas (Arb)	30	30
Aux gas (Arb)	5	5
Ion transfer tube temperature (°C)	320	320
Vaporizer temperature (°C)	300	300
MS1 Scan Range (Da)	84-1250	84-1250
MS1 Resolution	35000	35000
MS1 AGC target	1.00E+06	1.00E+06
MS2 Scan Range (Da)	84-1250	84-1250
MS2 Resolution	17500	17500
MS2 AGC target	2.00E+05	2.00E+05
Collision energy (CE)	30, 40, 50	30, 40, 50
Signal Intensity Threshold (cps)	1.00E+05	1.00E+05
Top N/Top speed	10	10
Exclusion duration (s)	3	3

Raw MS data were converted to mzXML format using ProteoWizard and processed with XCMS for peak detection, alignment, and retention time correction. MS1–MS2 feature matching was performed using precursor ion and retention time information, with mass and retention time tolerances of 25 ppm and 6 s, respectively. Peaks with a missing rate >50% across sample groups were removed, and the remaining missing values were imputed using the K-nearest neighbor (KNN) method. Peak areas were corrected using support vector regression (SVR). A pooled quality-control (QC) sample was prepared by mixing equal aliquots of extracts from all samples and was injected regularly, generally after every 10 analytical samples, to monitor analytical stability and reproducibility. Metabolite annotation was performed using an in-house high-resolution MS/MS database, public databases including METLIN, HMDB, KEGG, MoNA and MassBank, machine-learning-based in silico prediction, and metDNA. The comprehensive identification score integrated precursor ion matching, MS/MS spectral matching, and retention time filtering. For database searching, the precursor ion tolerance, MS2 tolerance, and retention time tolerance were set to 25 ppm, 50 ppm, and 60 s, respectively. Only metabolites with a comprehensive identification score >0.5 and a QC CV <0.5 were retained. Positive and negative ion-mode results were then merged, and duplicated annotations were resolved by retaining the entry with the highest annotation confidence and lowest QC CV.

Metabolite annotation confidence was reported using an MSI-style framework. Metabolites matched by accurate mass and MS/MS spectra against in-house or public databases were classified as Level 2, whereas metabolites annotated mainly by in silico prediction, metDNA, or compound-class information were classified as Level 3. No MSI Level 1 metabolites were reported in this study because authentic chemical standards were not used for confirmatory identification. Therefore, the annotated metabolites were regarded as putatively annotated compounds, and pathway interpretation was conducted cautiously.

### 2.5 Identification of differential metabolites

Differential metabolites (DMs) were identified based on the following three criteria: (1) a fold change (FC) of ≥ 2.0 or ≤ 0.5 (|log_2_FC| ≥ 1.0) in accumulation between the two accessions; (2) a variable importance in projection (VIP) score of ≥ 1.0 from the validated OPLS-DA model; and (3) an FDR-adjusted P value of ≤ 0.05. The VIP values were obtained from the validated OPLS-DA model and used to evaluate the contribution of each metabolite to group discrimination. A VIP threshold of 1.0 was applied because variables with VIP values greater than 1 are generally considered to have above-average importance in OPLS-DA models. To avoid dependence on the supervised model alone, VIP was used together with fold-change filtering and FDR correction.

### 2.6 KEGG pathway enrichment analysis of metabolites

Kyoto Encyclopedia of Genes and Genomes (KEGG) is a widely recognized and reliable database, an integrated pathway database and tool suite that links genomic and chemical information to molecular networks, supporting functional annotation and enrichment analysis (Kanehisa et al., 2012). Putative metabolite annotations were obtained by querying the KEGG Compound database, and the annotated features were further projected onto KEGG pathways for functional classification.

### 2.7 Statistical analysis

Data are presented as mean ± standard deviation (SD) of three biological replicates. Statistical analyses were performed using SPSS 22.0 (IBM Corp., Armonk, NY, USA). Before comparison between CK and 109, data normality and homogeneity of variance were assessed using the Shapiro–Wilk test and Levene’s test, respectively. For variables satisfying the assumptions of normality and equal variance, statistical differences between CK and 109 were assessed using Student’s t-test. When the assumption of equal variance was not met, Welch’s t-test was applied. A P-value of less than 0.05 was considered statistically significant. Figures were generated using OriginPro 2024b (Origin Lab Inc., MA, USA).

## 3 Results and discussion

### 3.1 Basic quality analysis of melons

As shown in **Table 3**, the fruit shape index of melon 109 was closer to 1.0 than that of CK, indicating a more spherical fruit shape. Fruit size and shape are critical quality traits in melon and other cucurbits, significantly influencing market classification and consumer preference (Amin et al., 2025). The average fruit weight of melon 109 was significantly lower than that of CK (*p* < 0.05), which is consistent with breeding objectives for fresh-market cultivars (Chi et al., 2025). From a practical perspective, the nearly spherical shape and reduced fruit size of melon 109 may facilitate grading, packaging, and retail display. Melon 109 exhibited significantly higher fruit firmness (8.5 ± 0.95 kg/cm²) than CK (*p* < 0.05), indicating a firmer texture that could enhance handling and shelf-life.

**Table 3:** The basic qualities of CK and 109 melons.

Cultivars	Fruit shape index	Single fruit weight (kg)	fruit flesh firmness (kg/cm^2^)	fruit flesh thickness (mm)	Total soluble solids (%)	Soluble sugar (%)	Titratable acid (‰)	Sugar-acid ratio
CK	1.20 ± 0.08a	2.55 ± 0.25a	3.87 ± 0.42b	37.08 ± 2.59a	11.63 ± 1.42b	16.49 ± 0.40a	0.64 ± 0.02b	256.87 ± 15.08a
109	1.06 ± 0.05b	1.14 ± 0.06b	8.50 ± 0.95a	29.41 ± 1.90b	16.24 ± 1.93a	18.21 ± 1.55a	1.37 ± 0.06a	132.88 ± 14.88b

Values are presented as mean ± SD (n = 3). Different lowercase letters indicate significant differences at *p* < 0.05.

The two accessions differed significantly in key compositional indicators associated with sweetness–acidity balance. Melon 109 showed a higher TSS value than CK (16.24% vs. 11.63%), exceeding the commonly reported range of approximately 8–14% for many melon cultivars (Kasampalis et al., 2025; Tzuri et al., 2025), and a 2.14-fold higher TA content, resulting in a markedly lower sugar–acid ratio. Notably, while TSS increased by approximately 40%, the soluble sugar content showed a relatively moderate increase of only about 10%. Because TSS reflects not only soluble sugars but also organic acids, amino acids, and other soluble metabolites, this quantitative discrepancy suggests that the elevated TSS in melon 109 is not attributable to sugars alone. From a fruit-quality perspective, the lower sugar–acid ratio of melon 109, together with its high TSS level, may indicate a more pronounced sweet–acid balance rather than a simple high-sugar phenotype. Previous sensory studies in melon have shown that consumer acceptance and flavor perception are associated with integrated sensory attributes and physicochemical traits, including sweetness, acidity, aroma, firmness, and soluble solids (Escribano et al., 2010; Farcuh et al., 2020). Therefore, the lower sugar–acid ratio of melon 109 may represent a potential quality-diversification trait for breeding melons with balanced sweet–acid flavor; however, it should not be interpreted as direct evidence of superior sensory quality because sensory evaluation was not performed in this study.

In conclusion, melon 109 exhibited higher firmness, higher TSS and TA, and a lower sugar–acid ratio than CK. These physicochemical differences suggest that the quality profile of melon 109 may be associated with coordinated changes in soluble metabolites, particularly sugars, organic acids, amino acids, and other soluble compounds. Therefore, untargeted metabolomics analysis was subsequently performed to identify the specific carbohydrate- and organic acid-related metabolites potentially associated with the elevated TSS, increased TA, and reduced sugar–acid ratio in melon 109.

### 3.2 Analysis of antioxidant indicators in melons

As shown in **Table 4**, melon 109 exhibited significantly higher ascorbic acid content and peroxidase (POD), catalase (CAT), and superoxide dismutase (SOD) activities, but significantly lower malondialdehyde (MDA) content than CK (*p* < 0.05). Ascorbic acid is an important water-soluble antioxidant in fruits and plays a central role in redox homeostasis, ripening regulation, and postharvest quality maintenance. Recent studies have also shown that higher ascorbic acid levels in melon are closely associated with improved antioxidant capacity and better quality retention during storage (Liao et al., 2023). MDA is widely regarded as a key indicator of membrane lipid peroxidation and oxidative damage. In this study, the MDA content of melon 109 was significantly lower than that of CK, indicating that 109 suffered less oxidative injury and maintained better membrane integrity. Similar findings have been reported in postharvest melon studies, where lower MDA accumulation was closely associated with delayed senescence and improved fruit quality (Wang et al., 2025).

**Table 4:** Antioxidant properties of CK and 109 melons.

Cultivars	Ascorbic acid content (mg/kg)	Malondialdehyde (mg/kg)	Peroxidase (U/g)	Catalase (U/g)	Superoxide dismutase (U/g)
CK	105.67 ± 12.82b	2.49 ± 0.02a	136.48 ± 3.56b	21.64 ± 0.68b	214.35 ± 4.63b
109	237.50 ± 5.56a	1.98 ± 0.01b	168.59 ± 4.24a	28.15 ± 0.71a	225.67 ± 1.33a

Values are presented as mean ± SD (n = 3). Different lowercase letters indicate significant differences at *p* < 0.05.

The activities of POD, CAT, and SOD were all significantly higher in melon 109 than in CK. These enzymes constitute the core enzymatic antioxidant defense system in plants. SOD catalyzes the dismutation of superoxide radicals into hydrogen peroxide, while CAT and POD further decompose hydrogen peroxide, thereby limiting excessive reactive oxygen species accumulation and alleviating oxidative stress. The coordinated increase in these enzyme activities in melon 109, therefore, suggests a more efficient ROS-scavenging system. Similar enhancement of antioxidant enzymes has been observed in melon under treatments that improve postharvest storability and oxidative balance (Dvořák et al., 2021).

In summary, melon 109 displayed a more favorable antioxidant-related physiological profile, featuring elevated ascorbic acid, higher activities of key antioxidant enzymes, and reduced MDA accumulation. These traits suggest a potentially stronger antioxidant-related status in melon 109, although direct ROS quantification and postharvest storage experiments will be required to confirm its functional significance.

### 3.3 Untargeted metabolomics analysis of melons

To further explain the biochemical basis underlying the observed differences in quality-related and antioxidant-related traits, untargeted metabolomics analysis was performed. UPLC–MS/MS analysis detected a total of 2101 metabolites in CK and melon 109, including carbohydrates, organic acids, amino acids and their derivatives, lipids, nucleotides, and secondary metabolites.

Analytical stability was evaluated using pooled quality-control (QC) samples in both positive and negative ion modes. The QC samples were prepared by mixing equal aliquots from all samples in the original analytical batch and were used only to monitor instrumental stability and analytical reproducibility. Because the QC pool included additional sample groups beyond the CK and melon 109 comparison analyzed in the present manuscript, its position in the PCA score plot was not interpreted as a biological intermediate between the two accessions. The TIC overlays and Pearson correlation heatmaps of QC samples showed consistent retention-time distributions, peak response patterns, and high correlations among QC injections, supporting the instrumental stability and analytical reproducibility of the UPLC–MS/MS detection process (**Supplementary Figures 1A–D**).

**Figure 2:**
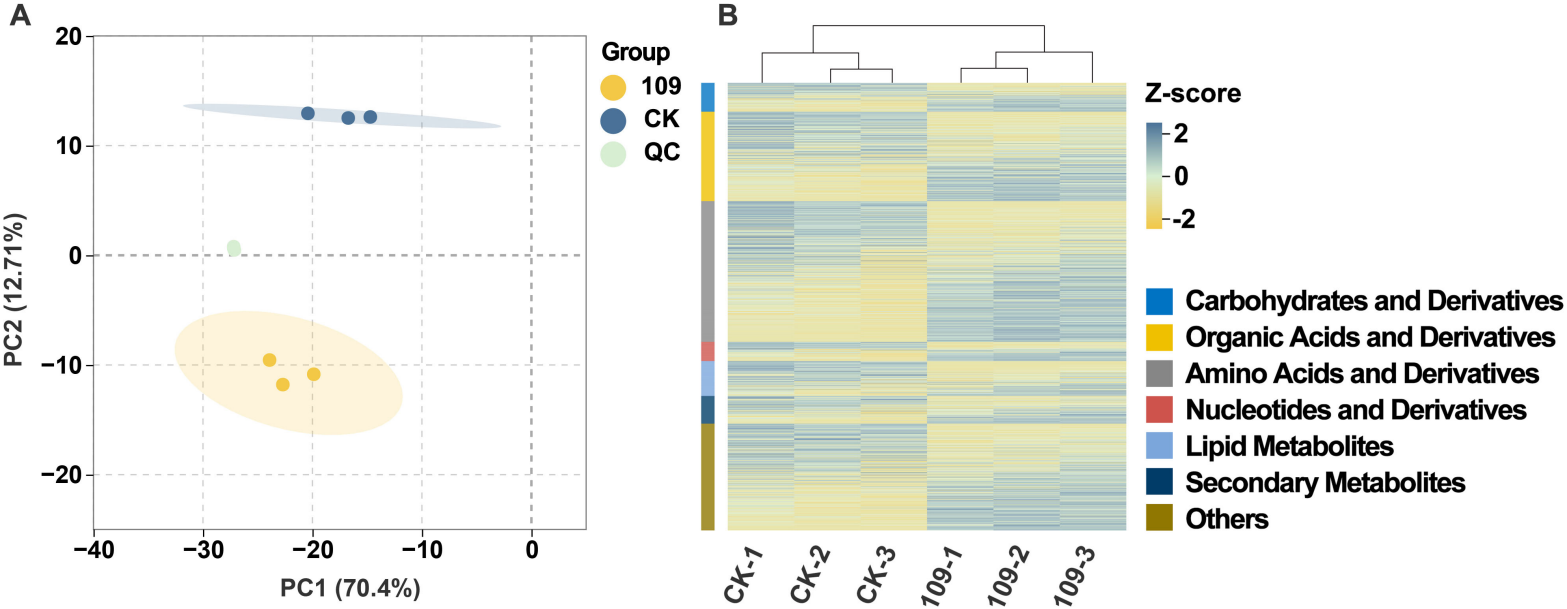
Principal component analysis **(A)** and hierarchical cluster analysis **(B)** of melon 109 and CK. Each sample had three biological replicates. The color from yellow (low) to blue (high) indicates the level of each metabolite. The Z-score represents the deviation from the mean by standard deviation units.

After QC-based filtering and signal correction, the metabolite peak areas were log10-transformed and scaled to unit variance before multivariate statistical analysis, a preprocessing strategy commonly used to reduce heteroscedasticity and balance the influence of high- and low-abundance metabolites (Wang K. et al., 2023). As shown in **Figure 2A**, PC1 and PC2 explained 70.40% and 12.71% of the total variance, respectively. CK and melon 109 were separated mainly along PC2, whereas PC1 appeared to reflect broader variation in overall metabolite abundance and pooled QC composition rather than the primary genotype-specific separation. The heatmap further showed that many metabolites exhibited higher normalized abundance in melon 109 than in CK, indicating a general increase in metabolite accumulation in melon 109. Hierarchical cluster analysis further separated CK and melon 109 into two genotype-specific clusters (**Figure 2B**), consistent with the PCA results.

### 3.4 Identification and classification of differential metabolites

To identify metabolites contributing to the metabolic differentiation between CK and melon 109, an OPLS-DA model was constructed and validated before differential metabolite screening. The OPLS-DA permutation test showed that the original model had high explanatory and predictive performance, with R²Y = 1 and Q² = 0.939. The permuted Q² values were generally lower than that of the original model, and the Q² regression line showed a negative intercept, indicating that the supervised model did not exhibit obvious overfitting (**Supplementary Figure 2**). Nevertheless, considering the limited number of biological replicates, the OPLS-DA model was used mainly as an auxiliary tool for feature prioritization rather than as the sole basis for biological inference.

**Figure 3:**
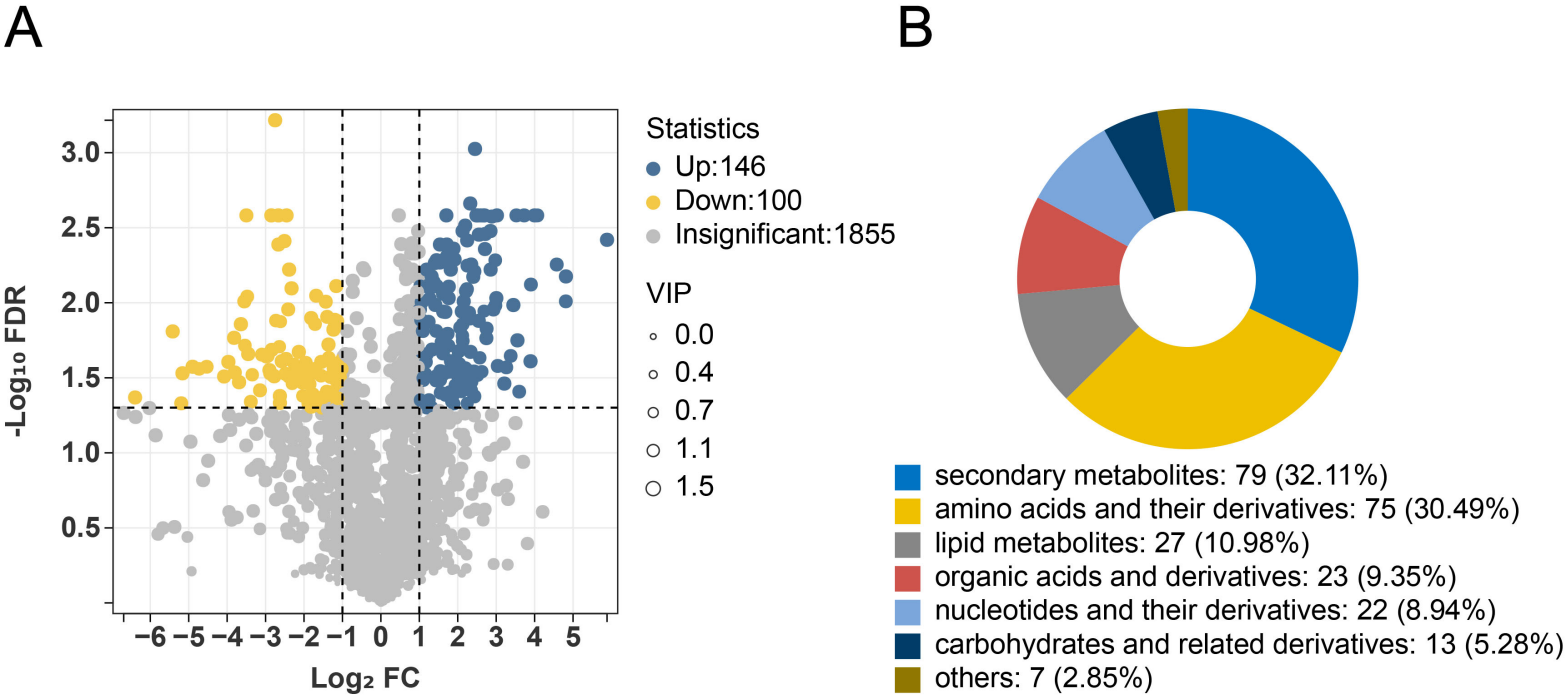
DMs between 109 and CK. **(A)** Volcano plot of the 2101 metabolites identified. **(B)** Pie chart of the biochemical categories of the 246 DMs.

Differential metabolites were screened using the combined criteria of |log_2_FC| ≥ 1, VIP ≥ 1.0, and FDR < 0.05. Based on these criteria, 246 differential metabolites (DMs) were identified between CK and melon 109, including 146 upregulated and 100 downregulated metabolites in melon 109, whereas 1855 metabolites were not significantly changed (**Figure 3A**).To clarify metabolite annotation confidence, annotation levels were summarized according to the Metabolomics Standards Initiative (MSI)-style reporting framework (Sumner et al., 2007). Among the 246 final DMs, 96 metabolites were annotated as MSI Level 2 (39.02%) and 150 metabolites as MSI Level 3 (60.98%). No MSI Level 1 metabolites were included because authentic chemical standards were not used for confirmation. These metabolites should therefore be regarded mainly as putatively annotated compounds rather than fully confirmed chemical identities, and individual metabolite interpretation was conducted cautiously.

The 246 DMs were assigned to seven biochemical categories (**Figure 3B**). Secondary metabolites represented the largest category, accounting for 79 DMs (32.11%), followed by amino acids and their derivatives with 75 DMs (30.49%), lipid metabolites with 27 DMs (10.98%), organic acids and derivatives with 23 DMs (9.35%), nucleotides and their derivatives with 22 DMs (8.94%), carbohydrates and related derivatives with 13 DMs (5.28%), and other metabolites with 7 DMs (2.85%). These results indicate that the metabolic differences between CK and melon 109 were not driven solely by primary metabolites, but reflected coordinated remodeling of primary-metabolism-related compounds and specialized metabolites.

### 3.5 Differential metabolites associated with compositional quality

To further explore the possible metabolite basis underlying the physicochemical differences observed between CK and melon 109, representative differential metabolites related to carbohydrates, organic acids, and amino acids were analyzed. In melon fruit, soluble sugars and organic acids are generally considered major contributors to sugar–acid composition (Cheng et al., 2022), whereas amino-acid-derived metabolites, secondary metabolites, and glycosylated compounds may provide putative clues related to flavor complexity, aroma potential, and sensory perception, although their direct sensory contribution requires further validation (Martínez-Rivas and Fernie, 2024).

**Figure 4:**
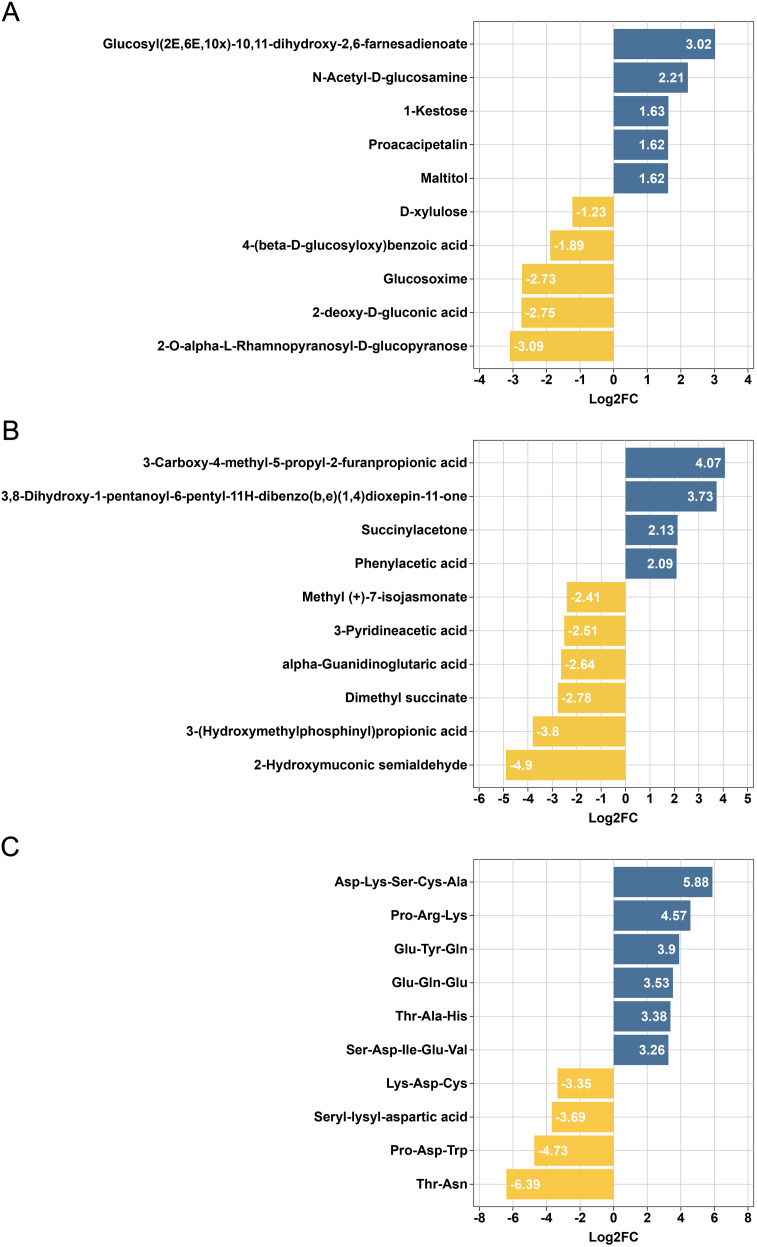
Bar plots of DMs of carbohydrates and derivatives **(A)**, organic acids and derivatives **(B)** and amino acids and derivatives **(C)**.

Among carbohydrates and related derivatives, the three most strongly upregulated metabolites in melon 109 were Glucosyl(2E,6E,10x)-10,11-dihydroxy-2,6-farnesadienoate, N-Acetyl-D-glucosamine, and 1-Kestose (**Figure 4A**). These compounds were not simple sweetness-determining sugars such as sucrose, glucose, or fructose, but were mainly glycosylated compounds, amino-sugar-related metabolites, or oligosaccharide-like carbohydrates. Glycosylated compounds have been proposed to serve as bound aroma-related reservoirs and may indirectly affect flavor complexity after hydrolysis (Liang et al., 2022; Chen and Quek, 2023). N-Acetyl-D-glucosamine-related metabolism may also be linked to N-glycan processing, which has been associated with fruit ripening and softening regulation (Ghosh et al., 2011), while 1-Kestose is a fructooligosaccharide component related to oligosaccharide metabolism (Tochio et al., 2018). Therefore, the carbohydrate-related changes observed in melon 109 may suggest broader remodeling of glycosylated and carbohydrate-derived metabolites rather than simple accumulation of soluble sugars.

Organic acids and their derivatives also showed marked differences between CK and melon 109. The three most strongly upregulated organic acid-related metabolites were 3-Carboxy-4-methyl-5-propyl-2-furanpropionic acid, 3,8-Dihydroxy-1-pentanoyl-6-pentyl-11H-dibenzo(b,e)(1,4)dioxepin-11-one, and Succinylacetone (**Figure 4B**). These metabolites may indicate changes in the organic-acid-related metabolite pool in melon 109. However, because they are complex organic acid derivatives rather than the major fruit acids directly responsible for titratable acidity, such as citric acid or malic acid, they should not be interpreted as direct determinants of TA. Instead, this pattern may suggest that the acidity-related phenotype of melon 109 is related to broader changes in acid-related and secondary metabolic pathways.

Among amino acids and their derivatives, the three most strongly upregulated metabolites were Asp-Lys-Ser-Cys-Ala, Pro-Arg-Lys, and Glu-Tyr-Gln (**Figure 4C**). These compounds were annotated as peptide-like amino-acid derivatives rather than free amino acids. Their accumulation may reflect changes in amino-acid-derived soluble metabolites and nitrogen-containing compounds in melon 109. Previous studies have suggested that amino acids, amino-acid derivatives, and peptides can contribute to taste background and flavor modulation in foods (Zhao et al., 2016).

Overall, the compositional differences between CK and melon 109 may be related to coordinated changes in carbohydrate-related metabolites, organic-acid-related derivatives, amino-acid-derived compounds, and specialized metabolites. These results are not sufficient to support a simple model in which primary metabolites alone explain the quality differences between the two accessions. Rather, they suggest that the higher TSS and TA of melon 109 may reflect broader changes in soluble metabolite composition, while glycosylated and secondary metabolites may contribute indirectly to flavor complexity or aroma potential. Further targeted quantification and sensory validation will be needed to confirm the direct contribution of these metabolites to sugar–acid balance and perceived fruit quality.

### 3.6 KEGG enrichment analysis of carbohydrate-, organic acid-, and amino acid-related DMs

To further explore pathway-level associations related to compositional quality, the DMs belonging to carbohydrates and related derivatives, organic acids and derivatives, and amino acids and their derivatives were selected for KEGG enrichment analysis. This subset included 111 DMs, comprising 13 carbohydrate-related metabolites, 23 organic-acid-related metabolites, and 75 amino-acid-related metabolites. KEGG enrichment analysis was performed with FDR correction, and pathways with FDR-adjusted p ≤ 0.05 were considered significantly enriched.

**Figure 5:**
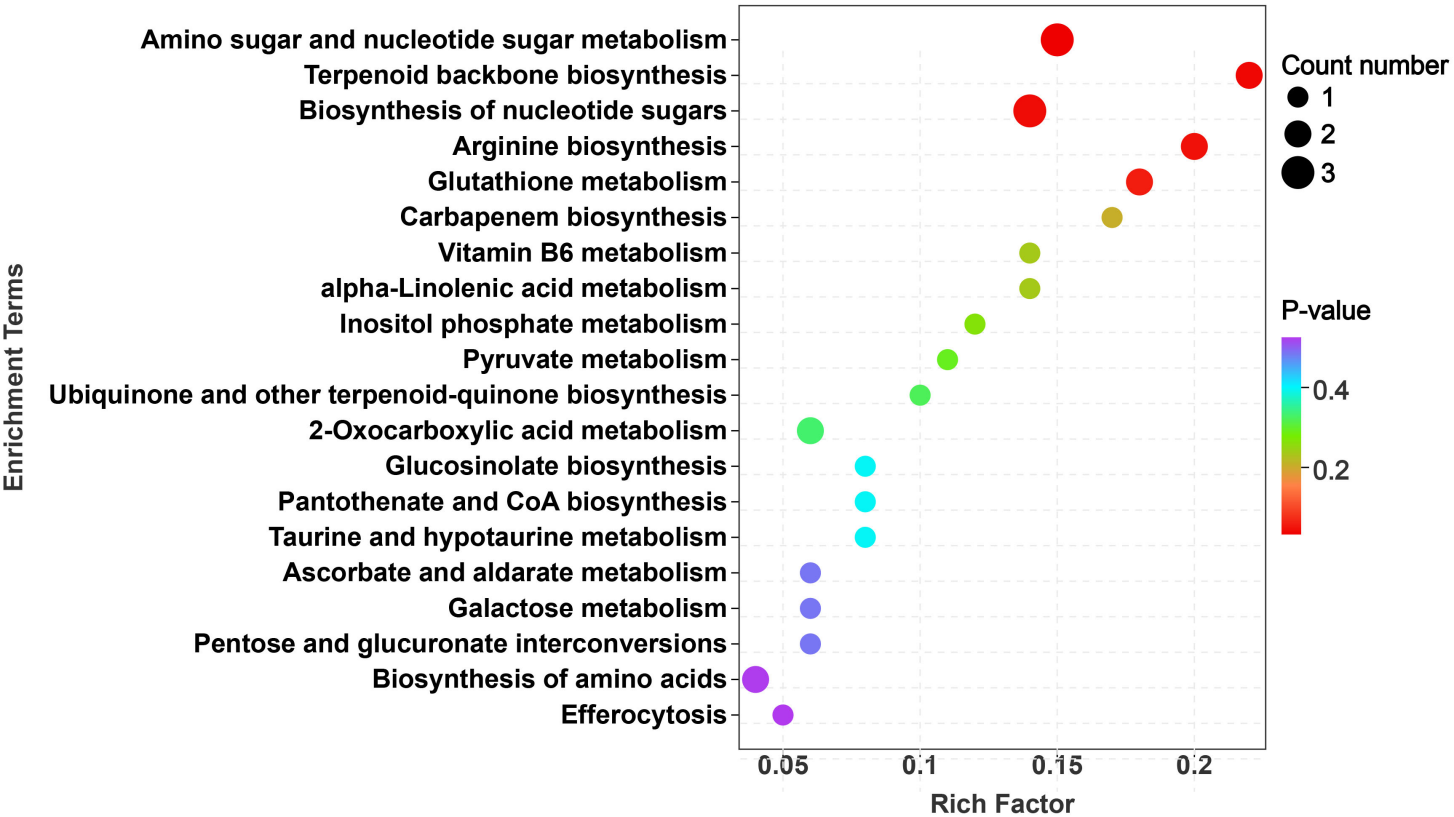
KEGG enrichment analysis of carbohydrate-, organic acid-, and amino acid-related differential metabolites.

**Figure 6:**
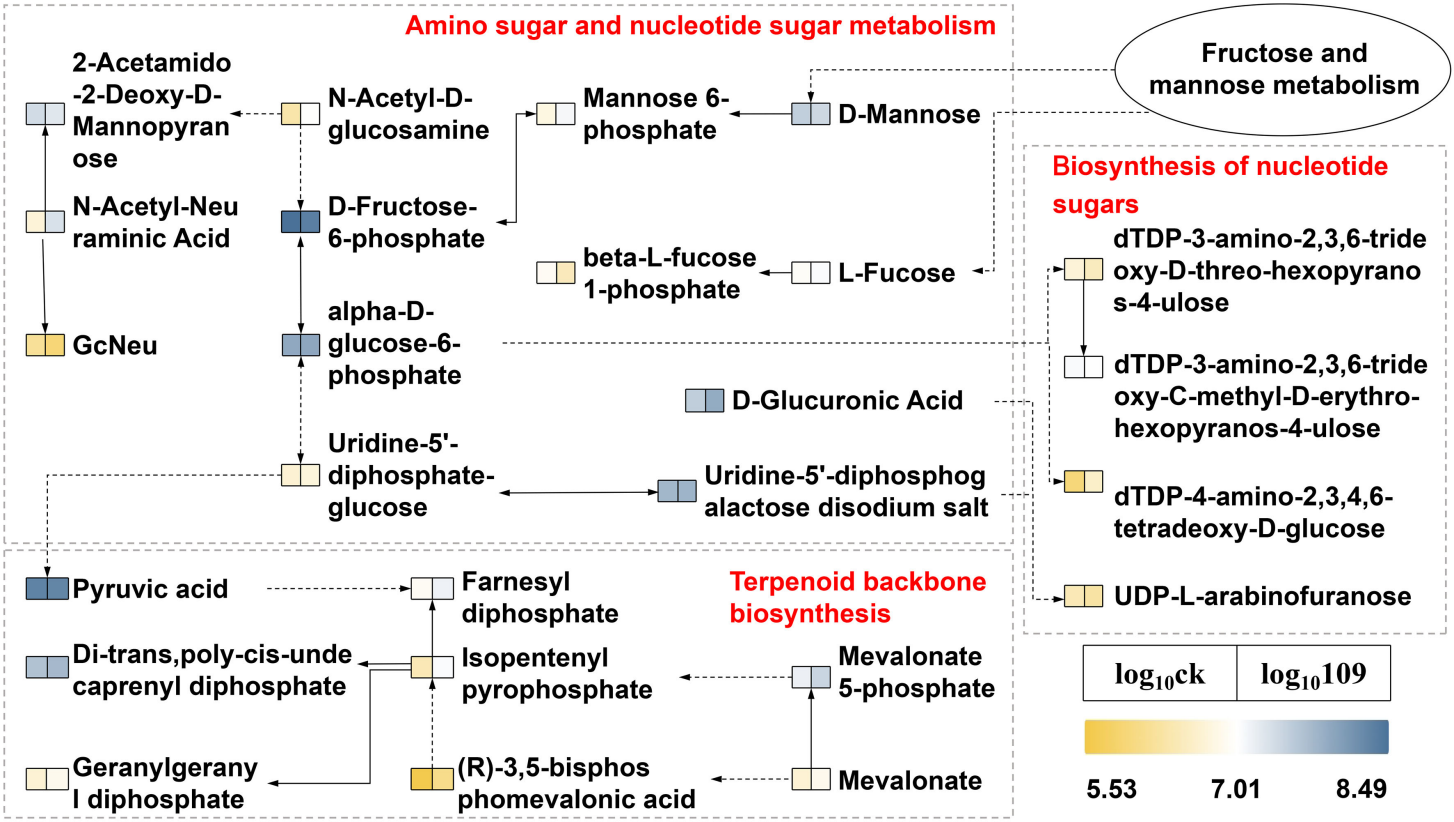
Maps of KEGG pathways involved in key differential metabolites. This map is constructed based on the KEGG pathway and literary references. The two adjacent boxes beside each metabolite represent log10-transformed abundance values in CK and melon 109, respectively; the color scale indicates abundance level. Solid arrows indicate direct or adjacent biochemical connections according to pathway annotation, whereas dashed arrows indicate indirect connections or omitted intermediate steps.

As shown in **Figure 5**, three pathways met the significance threshold: amino sugar and nucleotide sugar metabolism, terpenoid backbone biosynthesis, and biosynthesis of nucleotide sugars. To further visualize the metabolites mapped to these pathways, a schematic pathway overview was constructed (**Figure 6**). In the amino sugar and nucleotide sugar-related module, several metabolites, including N-Acetyl-D-glucosamine, N-Acetyl-Neuraminic Acid, and D-Glucuronic Acid, showed higher abundance in melon 109. N-Acetyl-D-glucosamine may indicate changes in amino-sugar-related metabolism and N-glycan-related processes, which have been associated with fruit ripening-related changes in previous studies (Irfan et al., 2022; Vare et al., 2025). D-Glucuronic Acid was interpreted more conservatively as a metabolite related to uronic-acid and nucleotide-sugar metabolism rather than as direct evidence of pectin remodeling, because pectin degradation is more directly associated with D-galacturonic acid-containing pectic polysaccharides (Gawkowska et al., 2018).

In the terpenoid backbone biosynthesis module, Mevalonate and Isopentenyl pyrophosphate showed higher abundance in melon 109. These metabolites are related to terpenoid precursor metabolism, and terpenoid-derived compounds are known contributors to fruit aroma formation (Lu et al., 2024). Therefore, this pathway may provide a clue to specialized metabolite remodeling and aroma-precursor potential in melon 109. However, this interpretation should be limited to pathway-level association, because volatile profiling and flux analysis were not performed.

Overall, KEGG enrichment analysis and pathway visualization suggest that the carbohydrate-, organic acid-, and amino acid-related DMs were mainly linked to amino-sugar metabolism, nucleotide-sugar metabolism, glycosylation-related carbohydrate conversion, and terpenoid-precursor-related metabolism. These results should be interpreted as pathway-level clues rather than definitive evidence of altered metabolic flux, enhanced aroma biosynthesis, or direct regulation of sugar–acid balance. Because sensory evaluation, volatile profiling, targeted functional assays, and metabolic flux analysis were not performed, the conclusions related to aroma potential, flavor perception, and metabolic fluxes remain putative and require further validation.

## 4 Conclusions

Melon 109 displayed a distinctive compositional quality profile characterized by higher flesh firmness, higher TSS and TA, a lower sugar–acid ratio, and enhanced antioxidant-related indicators compared with CK. These traits suggest that melon 109 may represent a useful germplasm resource for breeding melons with firmer texture, more pronounced sweetness–acidity balance, and improved antioxidant-related quality attributes.

The metabolomic results further suggest that the quality differentiation of melon 109 is unlikely to be explained by simple accumulation of soluble sugars or individual organic acids alone. Instead, it appears to involve broader remodeling of soluble metabolites, including carbohydrate-related derivatives, organic-acid-related compounds, amino-acid-derived metabolites, and specialized metabolites. In particular, the enrichment of amino sugar and nucleotide sugar metabolism, biosynthesis of nucleotide sugars, and terpenoid backbone biosynthesis points to possible involvement of glycosylation-associated carbohydrate metabolism and terpenoid-precursor-related metabolism in the compositional profile of melon 109.

The higher firmness observed in melon 109 may also be considered together with changes in amino-sugar- and glycan-related metabolites, such as N-Acetyl-D-glucosamine, although this relationship remains inferential because cell-wall composition and related enzyme activities were not directly measured. Similarly, D-Glucuronic Acid should be interpreted as a uronic-acid- and nucleotide-sugar-related metabolite rather than direct evidence of pectin remodeling. The terpenoid-related pathway results may indicate aroma-precursor potential, but they do not demonstrate enhanced aroma formation without volatile profiling.

Overall, melon 109 appears to possess a quality profile shaped by coordinated changes in physicochemical traits and multiple metabolite classes. These findings provide candidate metabolite classes and pathway-level clues for quality-oriented melon breeding, particularly for improving sweetness–acidity balance, texture, and aroma-related potential. Future work should combine targeted quantification of major sugars and organic acids, volatile profiling, sensory evaluation, cell-wall analysis, and multi-genotype validation to confirm the direct contribution of these metabolites and pathways to melon fruit quality.

## Data Availability

The datasets presented in this study can be found in online repositories. The names of the repository/repositories and accession number(s) can be found below: MetaboLights Accession REQ20260511219503.
