# Supplementary material for: Untargeted metabolomics reveals the metabolic basis of sugar–acid balance and quality differentiation in melon
Source: Front Plant Sci. 2026 Jun 19;17:1853789. doi: 10.3389/fpls.2026.1853789 (PMC13328509; doi:10.3389/fpls.2026.1853789)
Supplement: Supplementary file 1 [file Table1.docx]

Supplementary Material

# Supplementary Figures and Tables

For more information on Supplementary Material and for details on the different file types accepted, please see [here](https://www.frontiersin.org/guidelines/author-guidelines#supplementary-material).

## Supplementary Figures


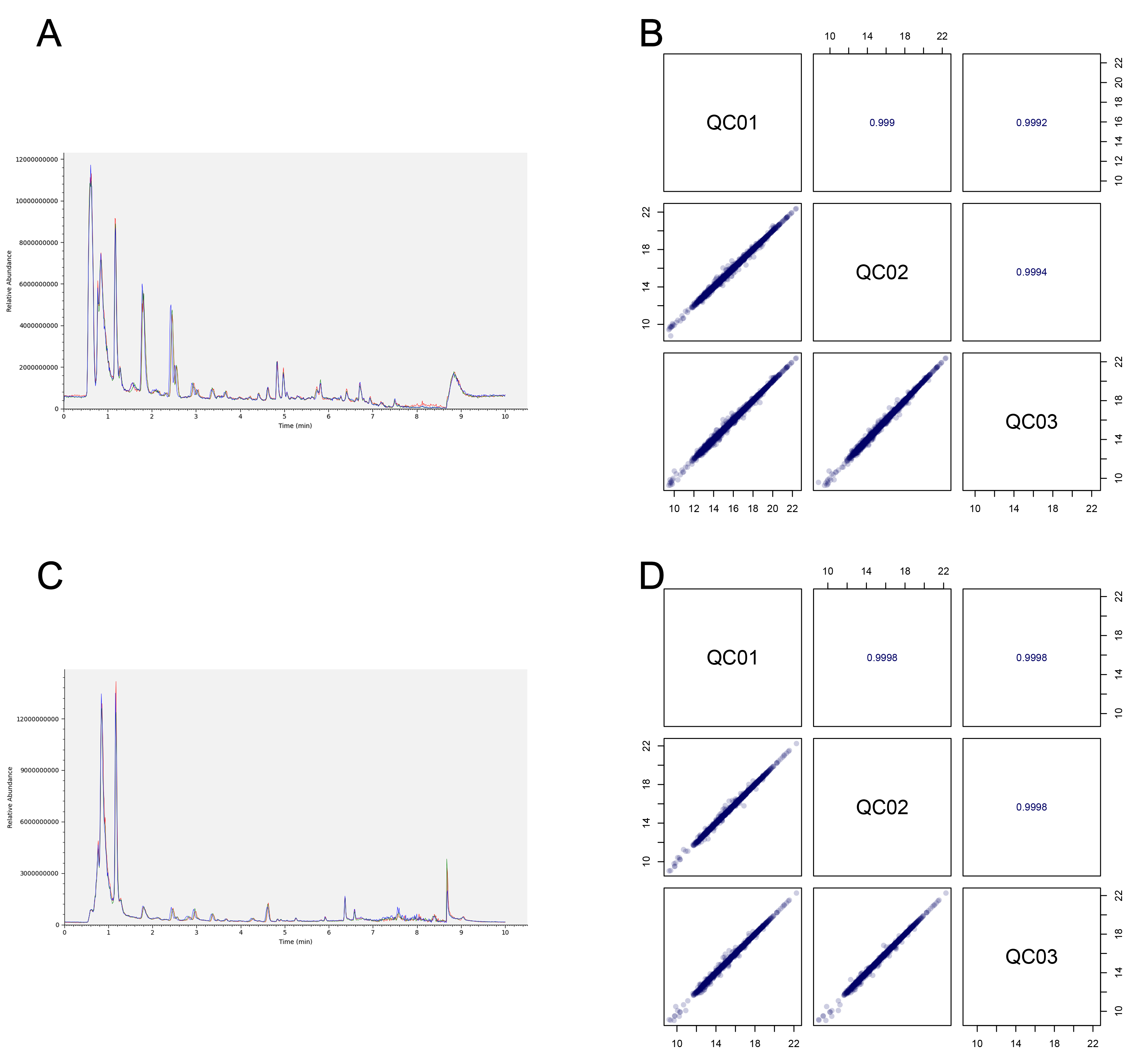


**Supplementary Figure 1.** Quality control evaluation of the untargeted metabolomics analysis.

1. Overlay of total ion chromatograms (TICs) of QC samples in positive ion mode. (B) Pearson correlation heatmap of QC samples in positive ion mode. (C) Overlay of TICs of QC samples in negative ion mode. (D) Pearson correlation heatmap of QC samples in negative ion mode.


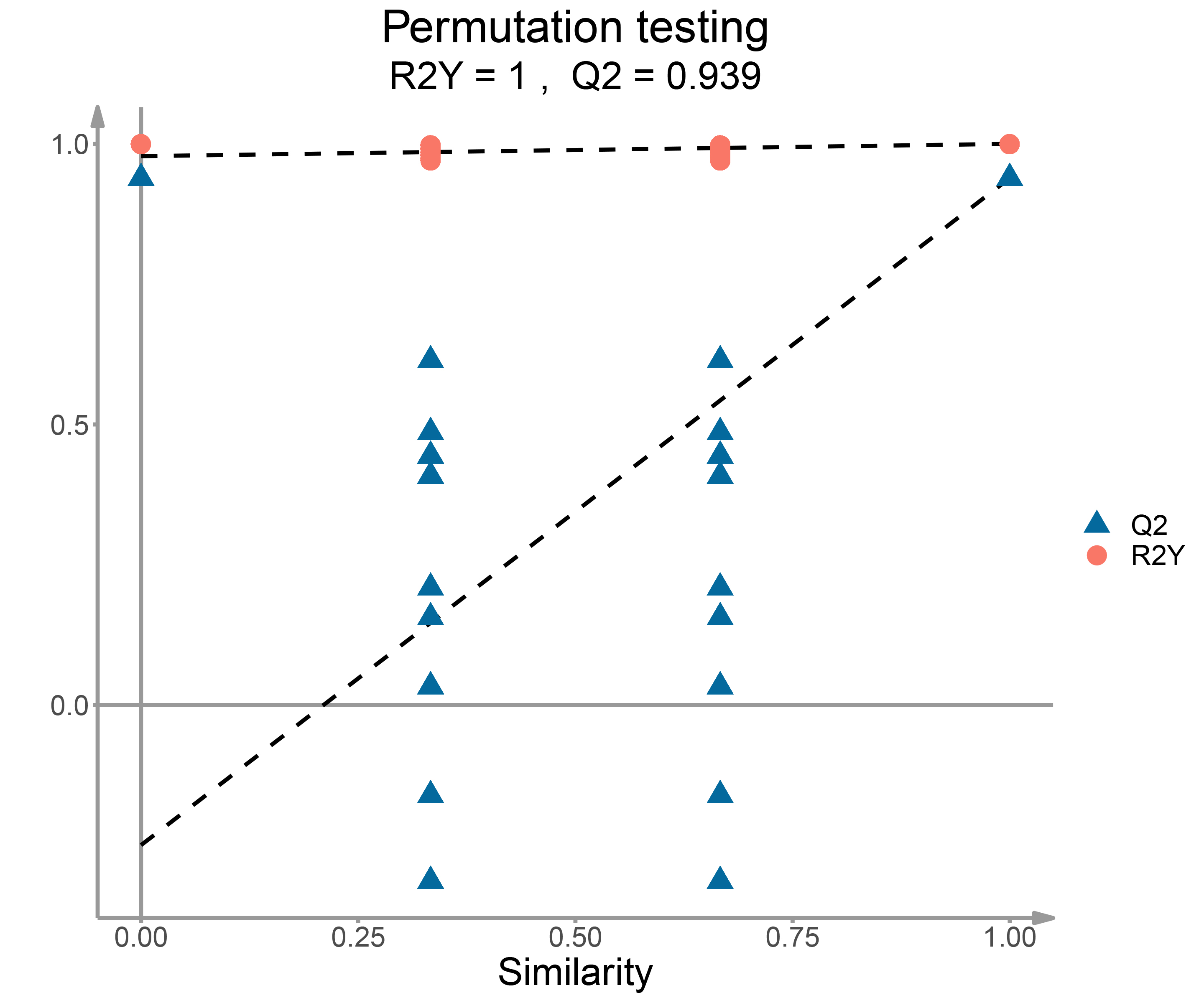


**Supplementary Figure 2.** OPLS-DA model validation by permutation testing.

The OPLS-DA model was validated using permutation testing. The original model showed R²Y = 1 and Q² = 0.939. The Q² values of the permuted models were generally lower than that of the original model, and the Q² regression line showed a negative intercept, suggesting no obvious overfitting.
